# Short stem total hip arthroplasty with the direct anterior approach demonstrates suboptimal fixation

**DOI:** 10.1007/s00264-020-04910-5

**Published:** 2021-01-11

**Authors:** Guido Garavaglia, Amanda Gonzalez, Christophe Barea, Robin Peter, Pierre Hoffmeyer, Anne Lübbeke, Didier Hannouche

**Affiliations:** Division of Orthopaedics and Trauma Surgery, Geneva University Hospitals, Geneva, Switzerland

**Keywords:** Total hip arthroplasty, Short stem, Loosening, Complications

## Abstract

**Purpose:**

Short stems use has increased substantially despite variable results reported in the literature. The purpose of this study was to report the rate of complications using a short stem implanted through the direct anterior approach (DAA), and to evaluate mid-term clinical and radiological results focusing on femoral stem fixation.

**Methods:**

Between April 2009 and November 2014, 698 elective total hip arthroplasties (THAs) were performed using a fully hydroxyapatite-coated short stem (AMIStem-H®). The mean age was 65.7 years (SD 12.6). Patients were invited for clinical and radiological evaluation, and to complete patient-reported outcomes questionnaires at two and five years after surgery. The mean follow-up was 6.2 years (range 2–9.73 years).

**Results:**

During the study period, 59 (8.5%) patients died and 24 (3.4%) were lost to follow-up. There were six (0.9%) dislocations and 12 (1.7%) fractures, seven occurred intra-operatively. Twenty-nine (4.2%) THAs required revision surgery. Eleven THAs were revised for aseptic loosening of the stem at a mean 4.9 years (1.2–7.3 years). Five years after surgery, radiographs of 324 THAs (324/425 eligible = 76.2%) were available. Stem subsidence ≥ 2 mm was present in 42 cases (12.9%), proximal radiolucencies in 101 hips (31.5%), cortical thickening in 52 (16.0%), and a pedestal in 219 (67.6%). An Engh score between − 10 and 0 was associated with lower HHS pain subscore (*p* = 0.005), a higher risk of stem revision for aseptic loosening (18.8% vs. 2.7%; *p* = 0.008), and was more frequent in younger patients with ASA score 1.

**Conclusion:**

Patients presenting radiological alterations at five years had an increased risk of revision for aseptic stem loosening and also inferior clinical results. Our study warrants further continued scrutiny of mid- and long-term survivorship of the AMIStem-H®, with radiological results at five years indicating suboptimal fixation of the stem in younger and active patients.

## Introduction

Short femoral stems might be an attractive option in young patients, as they preserve bone stock and supposedly allow more physiological loading on the proximal femur [1]. Short stem designs have been also developed to facilitate their insertion through less-invasive surgical exposures [2], including the direct anterior approach (DAA).

The AMIStem-H® (Medacta, Switzerland) is a cementless short femoral stem with a rectangular triple tapered design and hydroxyapatite (HA) coating. Based on straight rectangular tapered stems, it has been specifically designed for facilitating stem introduction through the DAA by reducing overall dimensions by 33% as well as the lateral flare of the stem. The AMIStem-H® is a type IIIB short stem according to the classification of Feyen et al. [3]. It can also be classified as a type 4 stem according to Khanuja et al. [4], or as a trochanter harming type according to Falez et al. [1]. In our institution, both the AMIStem-H® and the DAA were introduced simultaneously in 2008.

The purpose of this study was to assess the five year outcomes of the AMIStem-H® implanted through the DAA. We determined (1) the occurrence of short-term complications including revisions; (2) the clinical and patient-reported outcomes (PROs) based on the Harris Hip Score (HHS), the Short-Form health survey (SF-12®), the Western Ontario McMaster Universities (WOMAC) score, and the visual analogue scale (VAS) for evaluation of patients’ satisfaction; and (3) the radiological results with a focus on femoral stem fixation, bone ingrowth, and stress shielding; and (4) we evaluated the possible relation between patient demographics, femoral morphology, surgeon experience, and the primary fixation of the femoral stem.

## Methods

### Study design and population setting

A cohort study nested in a prospective hospital-based registry was conducted (IRB approval reference No. CER: 05-017 (05-041)). All consecutive patients operated between April 2009 and November 2014 through the DAA with the AMIStem-H®, an uncemented cup (Versafit®, Medacta, Switzerland) and a ceramic highly cross-linked polyethylene bearing were eligible for the study. All the procedures were performed on a traction table and were preceded by a thorough pre-operative planning to determine femoral neck angle, length, and offset. Thirty-five surgeons were involved in patient care. All procedures were performed by senior surgeons, or skilled physicians-in-training under direct supervision of a senior surgeon, as defined by the Accreditation Council for Graduate Medical Education. Exclusion criteria were as follows: THA performed for femoral neck fractures, or for pathological fractures secondary to cancer. Patients were followed until death, revision, or loss to follow-up. All patients had a minimum of two year clinical follow-up.

### Outcomes

The primary outcome was the occurrence of short-term complications, including intra- and post-operative fractures, dislocation, and infection, and all-cause revision.

Patients were invited for clinical and radiological evaluation at a five year follow-up visit. Additionally, a two year follow-up was performed in patients operated upon between April 2009 and December 2011. PROs were collected on average at one, two and five years after surgery and sent by mail to all patients still alive with a known valid postal address. Clinical evaluation and measures of PROs included the HHS, the SF-12, the WOMAC score, and the VAS (0–10) for evaluation of patient satisfaction (0 = lowest satisfaction; 10 = highest satisfaction). The measures were then converted to categorical data (dissatisfied, somewhat dissatisfied, somewhat satisfied, satisfied, very satisfied) according to Rolfson et al. [5].

### Radiological evaluation

All patients were followed radiographically pre- and post-operatively and at five years after surgery. Those operated between 2009 and 2011 underwent additional two year clinical and radiological controls. Radiographs were evaluated by an independent surgeon (GG) who did not participate in patient care. Femoral stem fixation, bone ingrowth, and stress shielding were assessed on digitized radiographs using specific templates and DICOMeasureTM software (ViewTec, Maison-Alfort, France). On the femoral side, we recorded the presence of focal osteolysis or radiolucent lines ≥ 1 mm in width in the seven zones of Gruen. We also measured the distance between the collar of the prosthesis and the lesser trochanter and between the tip of the greater trochanter to the shoulder of the prosthesis. Migration of the femoral stem was considered as definite, when there was a change in vertical distance of more than 2 mm [6]. The cortical index was calculated 10 cm distal to the lesser trochanter and the proximal femoral shape was recorded according to Dorr [7]. We also recorded the presence of cortical thickening and calcar hypertrophy or atrophy and the presence of a pedestal in zone 4. Stem fixation was evaluated using the Engh score (< − 10 = “unstable,” − 10–0 = “suboptimum but stable,” 0–10 = “ingrowth suspected,” > 10 = “bone ingrowth”) [6]. The Engh score was dichotomized in two categories: > 0 (categories “ingrowth suspected” and “bone ingrowth”) and ≤ 0 (categories “unstable” and “suboptimal but stable”). On the acetabular side, we examined the radiographs for cup migration, radiolucent lines, and osteolysis according to Charnley-DeLee zones.

### Covariates

The following covariates were routinely collected: gender, age, body mass index (BMI), ASA score, smoking status, education level, type of osteoarthritis (primary vs. secondary), femoral morphology, surgeon’s experience, surgery duration, head size, length of stay at the hospital, and discharge destination. Surgeon experience was classified according to the number of THAs performed and also categorized in < vs. ≥ 50 THAs with DAA performed. Among the 698 THAs, 493 (70.6%) were performed by experienced surgeons, who operated each more than 50 hips (Table 1).

**Table 1:** Baseline characteristics (*n* = 698 primary elective THAs)

	*N* (%)/mean
Women (%)	370 (53.0)
Age at operation (years), mean (SD)	65.7 (± 12.6)
Age in categories (%)	
< 55	135 (19.3)
55–64.9	156 (22.3)
65–74	221 (31.7)
≥ 75	186 (26.6)
Pre-operative BMI, mean (SD)	27.1 (± 5.0)
BMI in categories (%)	
< 25	240 (34.4)
25–29.9	298 (42.7)
30–34.9	115 (16.5)
35–39.9	32 (4.6)
≥ 40	13 (1.9)
ASA score (%)	
1	97 (13.9)
2	529 (75.8)
3	72 (10.3)
Smoking status (%)^1^	
Never smoker	417 (60.7)
Former smoker	132 (19.2)
Current smoker	138 (20.1)
Diagnosis (%)	
Primary OA	602 (86.2)
Secondary OA	96 (13.8)
Aseptic necrosis	44
Dysplasia	30
Inflammatory arthritis	11
Post-traumatic	7
Other	4
Surgeon experience, performed THAs > 50 (%)	493 (70.6)
Operation time (min), mean (SD)^2^	81.3 (± 27.8)
Head size (%)^3^	
28 mm	344 (49.4)
32 mm	314 (45.1)
36 mm	39 (5.6)
Mean FU time, months (range)	62 (1–93)

^1^Missing information on smoking status for 11 patients

^2^From the initial incision to final closure

^3^Missing information on head size for 1 patient

### Statistical analysis

Categorical variables were expressed as proportion, and for continuous variables, mean, standard deviations and ranges were reported. Cumulative incidence of all-cause revision was assessed using Kaplan-Meier survival analysis. Censoring was performed for death, lost to follow-up, or end of study. The actual duration of follow-up was considered for these analyses.

To compare clinical scores and PROs at two and five years, we reported (1) the results of all patients, who responded; and (2) only the results of those who had responded both at two and at five years. We used the Wilcoxon signed rank test for continuous variables with nonparametric distribution and the paired sample *t* test for continuous variable parametric distribution. For categorical variables, the McNemar-Bowker test was employed. To assess whether a lower Engh score (≤ 0) at two years was associated with an increased risk of aseptic stem loosening, we used Fisher’s exact test and also calculated a relative risk with 95% confidence intervals. To compare the level of pain on the HHS pain subscore at five years by the Engh score (dichotomized), we used the chi-square test (linear by linear association). To evaluate the association between the Engh score (dichotomized) and potentially influencing patient- and surgery-related factors, uni- and multivariable logistic regression models were used.

To evaluate inter-rater reliability of the Engh score, 50 interventions were selected randomly and the radiographic analysis was repeated by a second experienced reviewer (DH). The intraclass correlation coefficient (ICC two-way random) and 95% CI was used to quantify the inter-observer reliability of the Engh score (continuous) and the kappa statistic was used to quantify the inter-rater reliability of the dichotomized Engh score (> 0 vs. < = 0) [8].

The IBM® SPSS® Statistics version 22.0 software (IBM SPSS, Chicago, IL) and STATA version 11 were used for statistical analysis.

## Results

**Fig. 1:**
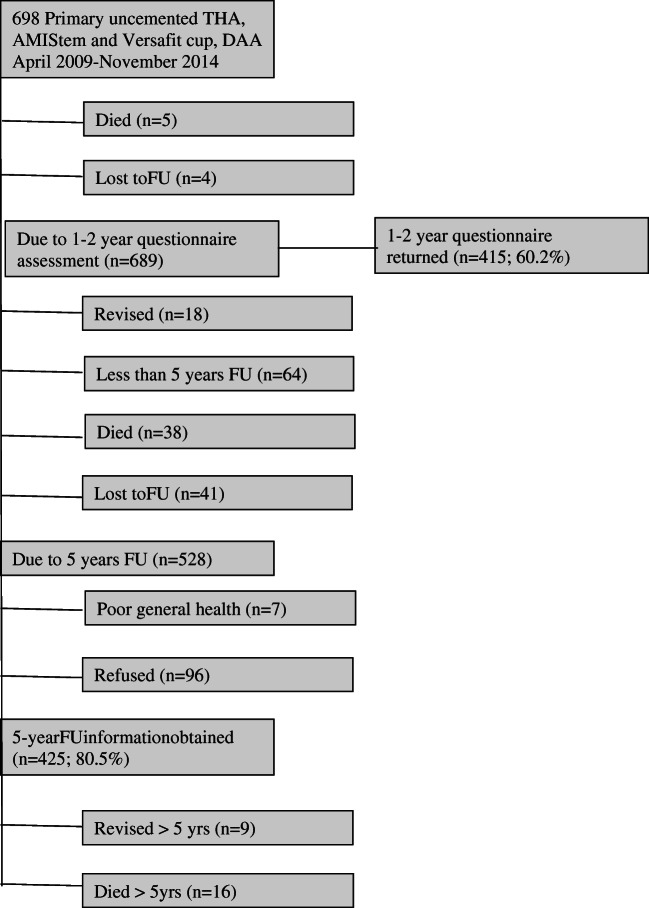
Flowchart

Overall, 698 THAs met the inclusion criteria and were included for the final analysis. For comparison, during the same period, a total of 1878 THAs (mean age 67.5 years, SD 13.1, 56% in women) were performed in our department for the same indications. For the current series, the mean age was 65.7 (SD 12.6, range, 18–96), 53% were women, and pre-operative BMI was 27.14 (SD 5, range, 17.3–48.9) (Table 1). The mean follow-up was 6.2 years (SD 1.8, range, 2–9.7 years). Over the entire follow-up period, 59 (8.5%) patients died and 45 (6.4%) were lost to follow-up (Fig. 1).

### Short-term complications including revisions

**Fig. 2:**
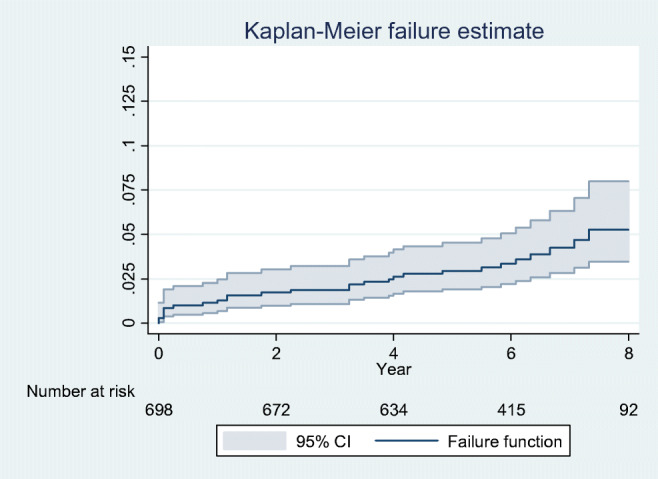
Kaplan-Meier failure function of Versafit cup/AMIS stem uncemented

Twelve (1.7%) fractures occurred, seven of them intra-operatively (Table 2). There were six dislocations (0.9%), all occurred during the first six months, and nine infections (1.3%), five of which were recorded during the first year after surgery. Overall, 29 (4.2%) THAs required a revision surgery (mean time to revision 3.0 years, SD 2.5, range 0.1–7.3 years). The main reason for revision was aseptic loosening of the femoral component (11 cases), followed by infection (8 cases), periprosthetic fracture (5 cases), implant malpositioning (2 cases), impingement (1 case), dislocation (1 case), and unexplained pain (1 case) (Table 2). Five-year cumulative incidence of all-cause revision was 3.3% (95% CI 2.2–5.0) (Fig. 2).

**Table 2:** Complications and revisions (*n* = 698 primary elective THAs)

	*N* (%)
Peri-operative fracture	7 (1.0)
Post-operative fracture during study period	5 (0.7)
Dislocation within 6 months	6 (0.9)
Dislocation after 6 months	0 (0)
Prosthesis infection within 1 year	5 (0.7)
Prosthesis infection during study period	9 (1.3)
Revision during study period	29 (4.2)
Revision cause	
Aseptic loosening	11
Infection	8
Dislocation	1
Fracture	5
Impingement	1
Implant malpositioning	2
Unexplained pain	1

More than 1 revision diagnosis possible

Eleven patients required revision because of aseptic loosening. Mean age for these 11 patients was 58.4 years (SD 14.3, range, 40–81); mean BMI was 27.7 (SD 7.1, range, 22.8–40.7); 4 (44.4%) patients were women; four, three, and two patients had an ASA score of 1, 2, and 3, respectively.

### Clinical results and patient-reported outcomes

Clinical results as measured with the HHS and all PROs largely improved after the intervention (Table 3). Thirty-four patients (34/366, 9.3%) reported thigh pain at five years. All PROs except for the SF-12 mental component score were worse at five years compared to two years, looking at all responders and also looking only at those, who had responded both at two and five years. Patient satisfaction (very satisfied and satisfied) was 94.2% at two years and 86.8% at five years (*p* = 0.006).

**Table 3:** Clinical results and patient-reported outcomes before and 2 and 5 years after surgery

	Pre-operative	2 years	5 years
Harris Hip Score*	*n* = 628	*n* = 115	*n* = 366
Total (SD)	50.9 (16.0)	92.2 (11.7)	90.4 (13.8)
Pain subscore, mean, SD	15.2 (8.7)	41.2 (5.8)	39.8 (8.1)
WOMAC pain, mean, SD	*n* = 518	*n* = 415	*n* = 364
	41.3 (19.0)	86.2 (18.8)	82.5 (22.3)
WOMAC function, mean, SD	*n* = 506	*n* = 414	*n* = 330
	41.9 (19.2)	80.4 (21.8)	78.4 (22.8)
SF-12, mean, SD	*n* = 512	*n* = 408	*n* = 328
Physical component score	34.5 (7.9)	45.3 (9.2)	43.7 (9.8)
Mental component score	44.8 (11.2)	48.0 (10.5)	47.6 (9.5)
Satisfaction (%)		*n* = 438	*n* = 387
Very satisfied		309 (70.5)	231 (59.7)
Satisfied		104 (23.7)	105 (27.1)
Somewhat satisfied		17 (3.9)	42 (10.9)
Dissatisfied/somewhat dissatisfied		8 (1.8)	9 (2.3)
UCLA activity scale (%)	*n* = 295	*n* = 409	*n* = 308
1–4 (low activity)	214 (72.5)	158 (38.6)	69 (22.4)
5–7 (moderate activity)	72 (24.4)	168 (41.1)	182 (59.1)
8–10 (high activity)	9 (3.1)	83 (20.3)	57 (18.5)

*Harris Hip Score total 0–100 (best), pain subscore 0–44 (best)

### Radiological results

Of the 698 THAs that were included, 64 had less than five years of follow-up, 43 had died at the time of that invitation, 45 were lost, and 18 THAs had already been revised before five years, leaving 528 THAs that were contacted for the five year follow-up (Fig. 1). Of the 528 THAs, five year outcomes (any clinical, X-ray, or questionnaire information) were obtained on 425 THAs (80.5%). Seven patients (1.3%) had poor general health and could not collaborate, and 96 patients (18.2%) refused the five year follow-up.

**Fig. 3:**
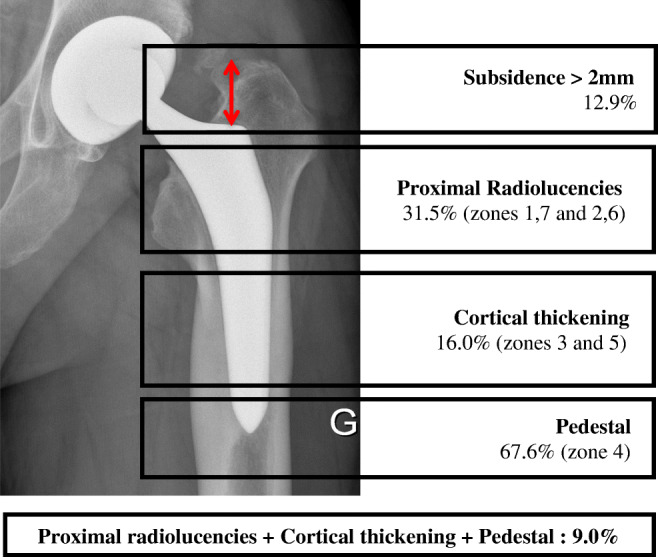
Distribution of detected radiological signs at 5-year FU

**Fig. 4:**
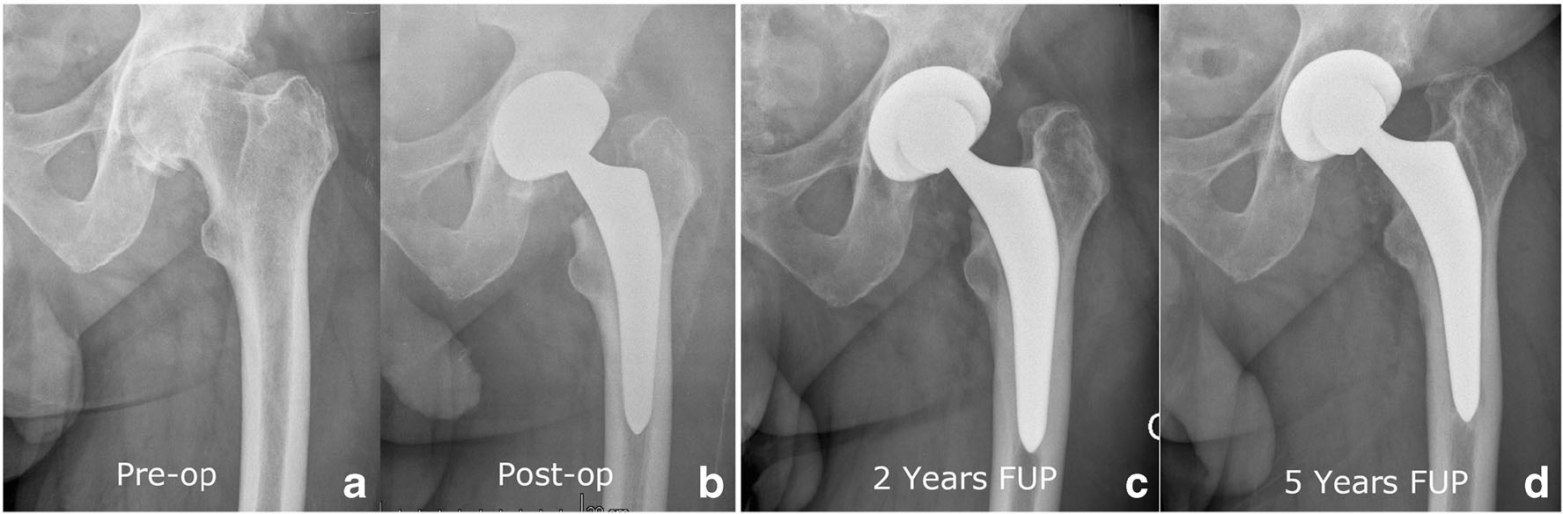
Illustrative case of aseptic loosening of the femoral stem of a 57-year-old patient who had a total hip replacement on his left hip. Radiological features of progressive loosening and mobilization of the femoral stem at two and five year follow-up. Joint aspiration was negative for bacteria as well as deep tissue cultures obtained from intra-operative samples. The hip was subsequently revised with a cemented femoral stem

Post-operative and five year radiographs (including those revised before 5 years) were available for 324 THAs (76.2%) (Table 4). There were no signs of cup migration nor loosening of the socket in all patients. Subsidence of the femoral component > 2 mm was seen in 42 hips (12.9%). Stem subsidence was significantly greater with small stem sizes (*p* = 0.027). Proximal radiolucencies were present in 101 hips (31.5%), cortical thickening in 52 (16.0%), and a pedestal in 219 (67.6%); 29 hips (9%) had all three signs (Figs. 3 and 4).

**Table 4:** Radiological results 2 and 5 years after surgery

	2 years	5 years
Engh score (%)*	*n* = 163	*n* = 324
> 10	92 (56.4)	101 (31.2)
10 to > 0	55 (33.7)	141 (43.5)
0 to > −10	15 (9.2)	80 (24.7)
≤ − 10	1 (0.6)	2 (0.6)
Radiolucent lines (RLL)	*n* = 163	*n* = 324
Yes (%)	25 (15.3)	101 (31.5)
Gruen zones 1/7**	34	201
Gruen zones 2–6**	6	34
Stem migration	*n* = 167	*n* = 326
Mean in mm (SD)	0.7 (2.6)	0.5 (1.2)
≥ 2 mm (%)	30 (18.0)	42 (12.9)
Pedestal	*n* = 163	*n* = 324
Yes (%)	48 (29.4)	219 (67.6)
Cortical thickening	*n* = 163	*n* = 324
Yes (%)	19 (11.7)	52 (16.0)

*Engh score is calculated from radiolucent lines, stem migration, pedestal, and calcar resorption

**Since RLL can be present both in proximal and distal zones of Gruen, the number of cases is higher than all RLL cases

The inter-rater reliability (ICC) regarding the Engh score (continuous variable) at five years was 0.570 (95% CI 0.349–0.731) indicating moderate agreement. For the dichotomized Engh score, the inter-rater reliability (kappa) was 0.619 indicating substantial/good agreement. For 16 hips, the Engh score was between − 10 and 0 (“suboptimal but stable”) two years after surgery. Compared to patients with an Engh score > 0 at two years, their risk of future stem revision for aseptic loosening was significantly higher (3/16 = 18.8% vs. 4/147 = 2.7%; RR 6.9, 95% CI 1.7–28.3, *p* = 0.008).

**Fig. 5:**
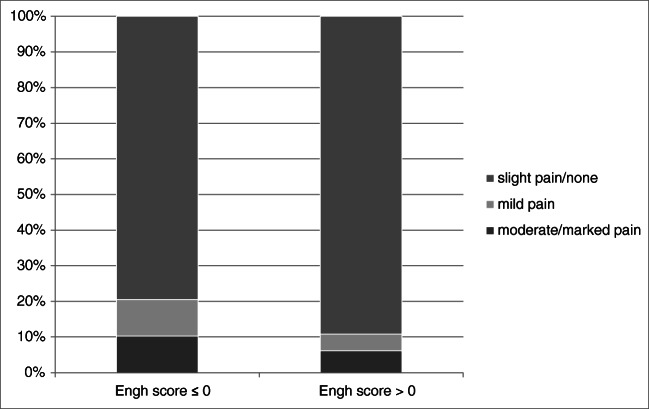
Harris Hip Subscore pain according to Engh score category. Engh score dichotomized > 0 and ≤ 0: < − 10 “unstable” and – 10–0 = “suboptimum but stable” vs. 0–10 = “ingrowth suspected” and > 10 = “bone ingrowth”; Harris Hip Score: 0–44 = no pain. When comparing HHS pain in Engh > 0 and ≤ 0, there were proportionally more patients having a low HHS pain in the ≤ 0 group. *p* = 0.037 chi-square linear by linear

When comparing HHS pain subscore at five years according to the Engh category > 0 vs. ≤ 0, significantly more patients had occasional, mild, or moderate-to-marked pain in the Engh category ≤ 0 group (*p* = 0.005) (Fig. 5). Thigh pain was also significantly more frequent in the Engh category ≤ 0 group (42.3% vs. 23.9%; *p* = 0.039).

### Patient demographics, femoral morphology, surgeon experience, stem size, and stem fixation

In univariate analysis, age < 55 years (*p* < 0.001) and having ASA score 1 (*p* = 0.048), increased the risk of being in the Engh score category ≤ 0. The association with ASA 1 was attenuated after simultaneously adjusting for age, which remained a strong predictor (*p* = 0.001) (Table 5). No association was found between the Engh score category and gender (*p* = 0.826), BMI (*p* = 0.805), surgeon experience (*p* = 0.828), stem size (*p* = 0.751), and Dorr type (*p* = 0.146).

**Table 5:** Association between patient- and surgery-related factors and Engh score < 0

	Univariable logistic regression	*p* value	Multivariable logistic regression	*p* value
	OR (95% CI)		OR (95% CI)	
Age at surgery, in categories		0.004		0.012
< 55 years	4.32 (1.91; 9.76)	< 0.001	3.93 (1.71; 9.05)	0.001
55–64.9 years	2.21 (0.96; 5.10)	0.064	2.12 (0.92; 4.93)	0.800
65–74 years	1.99 (0.90; 4.39)	0.088	1.94 (0.88: 4.29)	0.100
≥ 75 years	Reference		Reference	
Men	Reference		-	
Women	1.01 (0.62; 1.65)	0.958	-	
ASA score				
1	1.88 (1.01; 3.52)	0.048	1.45 (0.75; 2.79)	0.268
2–3	Reference		Reference	
BMI at surgery, continuous	0.99 (0.94; 1.03)	0.559	-	
Diagnosis			-	
Primary OA	Reference			
Secondary OA	1.25 (0.66; 2.37)	0.498		
AMIS stem size, continuous	0.98 (0.85; 1.13)	0.751	-	
Surgeon experience			-	
Less experienced (< 50)	Reference			
Experienced (> = 50)	1.26 (0.74; 2.15)	0.391		
Dorr classification			-	
Dorr A/B	Reference			
Dorr C	1.39 (0.78; 2.47)	0.264		

## Discussion

In the present study, we found a 4.2% revision rate, which was mainly due to aseptic loosening of the femoral component, and a high rate of patients presenting radiological features of poor stem stability. Our findings are comparable with the 2018 Australian implant registry data showing a 3.5% revision rate at five years for the AMIStem-H® combined with the same cup [9], but are higher than those reported for other short stems implanted through the DAA. In a retrospective review of 247 consecutive THAs with the Tribute® short stem, Attenello et al. [10] reported a femoral stem subsidence of more than 5 mm in four hips, and none required revision surgery at 27-month follow-up. In a retrospective review of 899 consecutive patients followed up to 24 months, Cidambi et al. [11] found a 1.3% revision rate for femoral aseptic loosening of a short, mediolaterally tapered stem. Interestingly, they found no cases of aseptic loosening in patients who received a standard-length collared, fully HA-coated stem. However, in a recent meta-analysis including 4280 patients with long stems and 2545 with short stems from 34 studies, Panichkul et al. [12] failed to find any difference in terms of revision for aseptic loosening between long and short femoral stems implanted through the DAA.

We found a periprosthetic femoral fracture rate of 1.7%, which is comparable to fracture rates reported with short stems implanted through the DAA [13]. In a case series of 640 patients, Dietrich et al. [14] reported a 1.6% femoral fracture rate when a short stem was used (130 Fitmore® stems and 53 hips AMIStems-H®) vs. 6.8% when a standard-length stem was implanted (457 Quadra® stems). The impact of prosthetic design and stem length on intra-operative femoral complications remains, however, unclear in the literature. Using a cementless standard-length femoral component, Jewett et al. [15] reported a high rate of intra-operative complications including 2.3% of trochanteric fractures, and 0.49% of femoral perforations or fractures. In contrast, in a retrospective series of 686 patients (851 hips), the use of short cementless tapered-wedge stems was associated with an increased 2.7% rate of fracture and was the only predictor for peri-operative periprosthetic complications [16].

Clinical results were globally very good with evident improvement of the HHS and WOMAC scores, and a high satisfaction level. However, comparing the satisfaction rate clinical scores and PROs between two and five years, we found a substantial increase in the proportion of unsatisfied patients, a decrease in the proportion of pain-free patients as well as lower WOMAC and SF-12 physical component scores five years after surgery.

Because radiological signs are so far the best predictor of implant survival, much of our attention was given to radiological results. We found femoral stem subsidence > 2 mm in 42 hips (12.9%), proximal radiolucencies in 101 hips (31.5%), cortical thickening in 52 (16.0%), and a pedestal in 219 (67.6%). To further evaluate femoral stem fixation, we used the Engh score and found a clear correlation between Engh’s stability categories and aseptic loosening. The calculated risk of stem revision for aseptic loosening was significantly higher for the hips with an Engh score between − 10 and 0 (category “suboptimal but stable”) when compared to hips with an Engh score > 0. We also found a correlation between reported pain and Engh score < 0, which suggests that these patients have not only an increased risk for aseptic loosening but also inferior clinical results. In a retrospective study including 100 primary THAs, Maier et al. [17] reported the results of the Fitmore® stem at a mean of 3.3-year follow-up. Radiographic analysis was performed for 79 THAs, with 20 hips (25%) demonstrating radiolucencies < 2 mm, 50 hips (63%) demonstrating cortical hypertrophy, and 13 hips (17%) demonstrating both proximal radiolucencies and distal cortical hypertrophy. The authors concluded that in these cases, the stem was probably stabilized by a distal fixation. Ishi et al. [18] showed that poor radiographic outcomes of uncemented stems were associated with a high canal flare index (CFI) and insufficient proximal filling, especially in Japanese women with narrow femoral canals. Similarly, D’Ambrosio et al. [19] evaluated the influence of femoral morphology or femoral component filling on radiological outcomes following THA using a fully HA-coated femoral stem. In a series of 183 THAs, they found that femurs with either proximally flared or distally narrowed canals, or insufficient proximal filling, tend to have less favourable radiological outcomes. Successful osseointegration was obtained with a filling threshold greater than 70% at 2 cm below the lesser trochanter. The authors also found a correlation between a low CFR measured 2 cm above the lesser trochanter and high CFI, which is more frequently associated to Dorr type A morphology [20]. In the present study, we did not evaluate the CFR, but we examined femoral morphology according to the Dorr classification. Interestingly, we did not find any correlation between the Dorr type and the occurrence of radiological changes or the Engh score category.

Finally, we found a clear correlation between patient activity and the Engh score: high activity, younger age, and lower ASA score were all associated with an Engh score < 10 (suboptimal fixation). In a series of 725 hip with signs of femoral stem loosening, Munger et al. [21] concluded that increased activity in younger patients with unrestricted mobility was an important factor of aseptic loosening of the femoral component. Whether young, healthy and more active patients, through the advantage of a less traumatic DAA, undergo a more rapid rehabilitation process, which can result in micromotion at the bone-implant interface, cannot be excluded nor confirmed.

The current study has several limitations. First, the concomitant introduction of a new stem and the DAA is clearly a limiting factor for this study, whereas it is not possible to determine if the stem design or the surgical approach or both are the cause of our findings. To our knowledge, there are no reports describing the use of the AMIS-H stem through other surgical approaches, which could have been useful for comparison purposes. Several authors have raised concern about the DAA being a potential risk factor for early aseptic loosening of the stem, especially in the early experience [22]. However, we did not find in our study any relation between surgeon experience and aseptic loosening or radiological signs. Second, we present a nonrandomized, observational study, in which there might be residual confounding factors and potential selection bias. The DAA has gained popularity among surgeons because it spares the abductors and allows faster recovery with shorter length of stay. Thus, it could have been proposed to younger and active patients who wanted to recover quicker. However, the mean age was 65.7 and 47% of the patients were male in our series, which is comparable to other studies [23], and to the age and gender of all THAs performed during the same period in our department. Third, despite an active strategy to increase response rates (phone calls, letters, emails when available), 96 patients (18.2%) refused the five year follow-up and were thus unavailable at the time of the analysis. We acknowledge that missing data might impact the robustness of our results. Finally, the appropriateness of the Engh score could also be debated, as it was rarely used to evaluate shorter stems [16]. In our study, there was a moderate-to-good agreement between the reviewers regarding the Engh score, with an inter-rater reliability that was actually higher than previously published with this scale [24].

In conclusion, short bone-conserving femoral stems in THA have been designed to preserve proximal bone stock for potential revision surgery, but also to facilitate less-invasive surgical exposures and revision procedures. However, little data are available on their design rationale, fixation features, and clinical outcomes. We found a high incidence of hips presenting radiological signs of suboptimal fixation in young and active patients, with an increased risk of revision for early aseptic loosening. The short stem evaluated in this study has been subsequently abandoned in all patients and replaced by a conventional length stem.

## Data Availability

All data and material pertaining to the study are made available upon request.
